# Breaking down walls in prostate cancer with the MURAL collection of patient-derived xenografts

**DOI:** 10.1038/s41467-021-25783-1

**Published:** 2021-09-17

**Authors:** Charlotte L. Bevan

**Affiliations:** Department of Surgery and Cancer, Imperial College London, Hammersmith Hospital Campus, London, UK

**Keywords:** Cancer genomics, Cancer models, Targeted therapies, Prostate cancer

## Abstract

A bank of 59 well-characterised prostate cancer patient-derived xenografts was established, including 17 classed as research-ready covering the disease-spectrum which, plus associated resources (organoids, serum, DNA/RNA profiles, tissue), are available for collaborative projects. This eagerly-anticipated resource will facilitate pre-clinical prostate cancer therapy studies.

Prostate cancer is the second most commonly diagnosed male cancer globally and a leading cause of cancer death^1^, despite a plethora of available and emerging therapies and the best research efforts of hundreds of labs worldwide. Reliable and representative pre-clinical models are urgently needed, to facilitate pre-clinical translational and mechanistic studies of new and combination therapies. The report from Risbridger, Clark, Porter et al, detailing establishment by the multi-centre MURAL (Melbourne Urological Research Alliance) team of a large bank of well-characterised and functional prostate cancer PDXs with associated molecular and clinical characterisation, capable of being transported and available to collaborators, will thus be greeted with enormous enthusiasm in the field^2^.

Although effective prostate cancer therapies exist, therapy resistance is almost inevitable so there is a continuing need to develop new options, preferably with associated predictive biomarkers. Another issue is high disease heterogeneity: unlike primary breast cancer, which has clear markers delineating luminal, A, luminal B, HER-2 enriched and triple negative tumours, defining the best treatment options, prostate cancer has at best been separated into 7 subclasses defined by genomic alterations, which are neither prognostic nor predictive^3^, although the PAM50 classifier approach has now been used to define, with analogy to breast cancer, subtypes also termed luminal A, B and basal that may be better predictive of therapy response^4^. All these factors make having robust, representative pre-clinical models paramount. Readily available, tractable cell lines are limited and the majority in use were derived from patients well before currently-used drugs (e.g. the new generation of antiandrogens such as enzalutamide, darolutamide, the steroid synthesis inhibitor abiraterone, even the widespread use of taxane chemotherapies^5^) were adopted and so using them to screen and study new drug treatments and drug resistance, while by no means impossible, has its drawbacks. Cell lines also lack many of the factors that can contribute to tumour phenotype—3D architecture, the presence of stromal cells, vasculature. Short-term cultures of biopsy material can address these issues to some extent^6^, but there are drawbacks to this also—potential loss of tumour architecture during culture, the inability to expand them severely limits the number of treatments possible, and they are highly heterogenous so there is often insufficient tumour tissue to adequately characterise them genetically or transcriptionally. Thus many would welcome the option to use patient-derived xenografts (PDXs), of which the gold standard is extensively characterised (genetically, epigenetically, transcriptionally), serially transplantable (for experimental expansion and longevity) yet relatively stable (genetically, epigenetically, transcriptionally) xenografts representing different ethnicities and different disease stages, including therapy resistance. The rates for successful engraftment of prostate cancer PDXs appear to be worse than many other tumour types and deriving such models is expensive, requiring an efficient pipeline with cooperation from patients and their families, and substantial infrastructure. It is therefore not feasible for many labs to establish their own PDXs, and the availability from commercial suppliers is very low—at the time of writing, the JAX® PDX database lists only two prostate cancer PDXs, compared to 44 breast cancer, 81 lung cancer and 16 pancreatic cancer PDXs (https://www.jax.org/jax-mice-and-services/in-vivo-pharmacology/oncology-services/pdx-tumors).

The accompanying paper describes 59 prostate cancer PDXs, derived from 41 tumours from 30 patients over 8 years^2^. It is important to note that these represent the successful implants from a total of 208 xenograft attempts from 88 patients. The resulting 59 PDXs represent treatment-naïve primary cancer, therapy-resistant cancer and metastases, also adenocarcinoma and neuroendocrine disease. Adenocarcinoma accounts for the vast majority of prostate tumours and is usually positive for expression of the androgen receptor (AR)—the key therapy target in prostate cancer. True de novo neuroendocrine prostate cancer, AR-negative and androgen-independent, is very rare and yet, in recent years, the increasing emergence of neuroendocrine-like tumour phenotypes late in disease course has been noted. Termed treatment-induced NEPC (tNEPC), these often do not express AR, and may be attributable to increased use of androgen ablation driving tumours to an androgen-independent or androgen-indifferent phenotype^7^. The MURAL collection contains 33 adenocarcinomas from 19 patients, 18 neuroendocrine tumours from 7 patients, and 8 “mixed” tumours, which have both AR expression and neuroendocrine markers.

Particularly exciting is the availability of matched primary and metastatic tumours from the same patient, and different metastatic sites from the same patient. It is ideal to be able to compare the response of primary vs metastasis, metastasis vs metastasis, originating from the same primary tumour so with the same originating genotype. The MURAL collection includes tumours established from a large range of different metastatic sites (not always with a matched primary), including lung, brain, lymph node, dura and liver. However, no PDXs were successfully established from bone metastases, which is disappointing given bone is the most common metastatic site in prostate adenocarcinoma^7^. The authors believe this to be due to difficulty in processing bone samples for sub-renal grafting, which is easier for soft tissues.

Further, to aid the investigation of drug resistance and treatment sequencing, 18 of the available PDXs are “castration resistant” derivatives (termed PDX Cx and derived by growth in castrated mice) that can be studied alongside their hormone-sensitive counterpart PDX. Castration resistance refers to resistance to any AR-inhibitory therapy (antiandrogens, pituitary downregulation, steroid synthesis inhibition) and is the most prevalent and pressing issue in management of prostate cancer^5^. Interestingly, in 2 cases, a PDX with a mixed pathology has given rise to a Cx subline with a neuroendocrine phenotype, and in another case an adenocarcinoma PDX gave rise to a mixed phenotype Cx, supporting the theory that androgen ablation can promote emergence of neuroendocrine disease. However, this cannot be generalised since in 8 cases the Cx PDX derived from an adenocarcinoma PDX retains the adenocarcinoma subtype. Studying such paired models could be instrumental in delineating the molecular mechanisms of emergence of tNEPC.

Possible mechanisms for development of castration resistance was investigated by comparing genetic profiling (targeted DNA sequencing) of the matched pairs. This revealed higher frequency of genome alteration in metastasis-derived PDXs and a pattern of genomic alterations comparable to that seen in other studies of the genetic landscape of advanced/metastatic prostate cancer^8–10^, with TP53, RB1, PTEN, MYC being the most frequently altered and AR itself ranking 11th. Interestingly, when comparing castration-resistant adenocarcinomas to their hormone-sensitive counterparts, even in the AR-positive tumours there was little change in AR copy number (a non-significant increase in 2 cases) and no additional AR mutations. It would be of interest to know whether AR transcript levels and/or activity are increased by other means; it could also be the case that pre-existing mechanisms of resistance arose before the engraftment of the original PDX, negating further amplification of AR signalling.

This MURAL collection also contains associated available resources that include organoids, mouse serum, DNA and RNA profiles, tissue microarrays and clinical data (including biomarker expression) plus fresh, fixed and frozen tissue samples. The availability of characterised, transportable organoids will expand the use of these pre-clinical models. 22 of the 24 PDXs tested in this regard showed growth as organoids, to differing extents (some limited), and cryopreserved organoid cultures have been sent to other labs who have produced organoids from them^11^. This ability to grow as organoids is a feature of 14 of the 17 research-ready PDXs.

This is, however, one of a number of PDX collections. The largest are the LuCaP series of 21 PDXs established by Eva Corey and co-workers (several of which also have castration-resistant derived PDXs)^12^ and the MDA PCa series at MD Anderson, of which 47 PDXs can be expanded as cell lines as well as PDXs^13^. These and others supported by Movember’s GAP (Global Action Plan) xenograft initiative, totalling 98 PDXs of which 15 are derived castrate-resistant clones, were recently summarised^14^, These collections are dynamic and growing, while smaller collections and individual reports of new PDXs are increasingly evident in the literature. Perhaps the most pressing unmet need now in the area of PDXs is—given the acknowledged health disparities in prostate cancer—for them to better reflect different ethnicities. Few papers address this directly and the MURAL study is no exception. The MD Anderson collection is derived from donors of whom the racial distribution reflects their patient population (88 Caucasian, 6 African American, 5 Hispanic) and two of the 47 PDXs that can be readily expanded are from the same African American donor while 4 others are derived from 2 Hispanic donors^13^.

In summary, the MURAL collection is notable for the inclusion of primary-metastatic and metastatic-metastatic pairs, for the wealth of associated data and resources perhaps most outstanding being the organoids, and the focus on ease of sharing. Research-ready PDXs and organoids have already been successfully distributed (cryopreserved^15^) to collaborating labs and will be made available for peer-reviewed, ethically approved projects. Further, the detailed methods and supplemental data will improve chances of success for labs who aim to derive their own pipelines to PDXs. I look forward to seeing advances in prostate cancer research made by labs the world over, facilitated by access to this invaluable resource.
